# The Association Between the Digital Divide and Health Inequalities Among Older Adults in China: Nationally Representative Cross-Sectional Survey

**DOI:** 10.2196/62645

**Published:** 2025-01-15

**Authors:** Mengqiu Wu, Yongxi Xue, Chengyu Ma

**Affiliations:** 1 Key Laboratory of Carcinogenesis and Translational Research (Ministry of Education/Beijing) Department of Genetics Peking University Cancer Hospital & Institute Beijing China; 2 School of Public Health Capital Medical University Beijing China

**Keywords:** older adults, digital divide, internet use, internet access, health inequalities

## Abstract

**Background:**

Health inequalities among older adults become increasingly pronounced as aging progresses. In the digital era, some researchers argue that access to and use of digital technologies may contribute to or exacerbate these existing health inequalities. Conversely, other researchers believe that digital technologies can help mitigate these disparities.

**Objective:**

This study aimed to investigate the relationship between the digital divide and health inequality among older adults and to offer recommendations for promoting health equity.

**Methods:**

Data were obtained from the 2018 and 2020 waves of the China Health and Retirement Longitudinal Study. Physical, mental, and subjective health were assessed using the Activities of Daily Living (ADL) scale, the Instrumental Activities of Daily Living scale, the Mini-Mental State Examination scale, and a 5-point self-rated health scale, respectively. The chi-square and rank sum tests were used to explore whether internet use and access were associated with health inequality status. After controlling for confounders, multiple linear regression models were used to further determine this association. Sensitivity analysis was conducted using propensity score matching, and heterogeneity was analyzed for different influencing factors.

**Results:**

The 2018 analysis highlighted widening health disparities among older adults due to internet access and use, with statistically significant increases in inequalities in self-rated health (3.9%), ADL score (5.8%), and cognition (7.5%). Similarly, internet use widened gaps in self-rated health (7.5%) and cognition (7.6%). Conversely, the 2020 analysis demonstrated that internet access improved health disparities among older adults, reducing gaps in self-rated health (3.8%), ADL score (2.1%), instrumental ADL score (3.5%), and cognition (7.5%), with significant results, except for ADL. Internet use also narrowed disparities, with significant effects on self-rated health (4.8%) and cognition (12.8%). The robustness of the results was confirmed through propensity score–matching paired tests. In addition, the study found heterogeneity in the effects of internet access and use on health inequalities among older adults, depending on sex, age, education, and region.

**Conclusions:**

The impact of internet access and use on health inequalities among older adults showed different trends in 2018 and 2020. These findings underscore the importance of addressing the challenges and barriers to internet use among older adults, particularly during the early stages of digital adoption. It is recommended to promote equitable access to the health benefits of the internet through policy interventions, social support, and technological advancements.

## Introduction

### Background

According to data from China’s National Bureau of Statistics, by the end of 2023, the population aged ≥60 years in China had surpassed 290 million. This aging trend exceeds the world average and continues to rise. In response, China has introduced a national strategy to address population aging during the 14th Five-Year Plan (2021-2025) period. Health inequalities among older adults are widespread across various age groups and social structures [1]. These disparities are influenced by factors such as individual characteristics, the distribution of medical resources, regional economic development, and public policies [1]. They include differences based on socio-economic status [2,3], sex [4], education [5], and area of residence [6].

Meanwhile, the rise of information and communication technology has introduced digital health as a substantially factor in improving health outcomes. According to findings published by the China Internet Network Information Center [7] in 2020, only 24% of the older adult population in China has adopted internet use. Older adults face substantial challenges in accessing digital resources compared to other demographics, leading to a new form of social structural inequality known as the “digital divide” [8]. This divide is more pronounced among older adults due to limited internet access, the complexity of operating digital devices, and slower acceptance of new technologies [9].

Previous research presents differing views on the relationship between health inequity and the digital divide. One perspective holds that digital technology can enhance health, particularly in remote areas, through telemedicine, optimizing resource distribution, and promoting health equity [8]. Conversely, another perspective suggests that the digital divide may widen health disparities between older and middle-aged adults as well as among different groups of older adults. Digital health technologies, such as electronic medical records, telemedicine, disease surveillance, diagnostics, and drug discovery, are well established [10]. However, barriers to online health care modalities among older adults exacerbate health disparities, affecting equity in health care use [11]. For instance, some researchers have highlighted that inequality in access to and use of digital health is one of the key factors contributing to increased mortality rates among older adults during the COVID-19 pandemic [12].

Most previous studies have focused on the impact of internet use on health, yet it remains unclear whether such use has widened or narrowed health inequalities among different older adult populations. Given the varied effects of internet use across different demographics and the scarcity of research on its relationship with health inequalities, this paper uses cross-sectional data from China to construct a health inequality index. The aim is to explore the correlation between the digital divide and health inequality among older adults, thereby addressing gaps in existing research and providing strategies for improving health equity for older adults. This paper contributes by exploring how internet technology can be leveraged to improve the health status and equity of older adults, ultimately providing effective solutions to enhance social equity and reduce the digital divide.

### Status of Health Inequalities Among Older Adults

Older adults bear a greater health burden as a group considered vulnerable, and health inequalities are more pronounced due to their living conditions and health-related behaviors. Health encompasses more than just the physical integrity of an individual [13]; it represents a comprehensive state of well-being requiring evaluation through various metrics. Health inequality indicators can be measured through subjective and objective measures. Subjective indicators are generally assessed by calculating the relative deprivation index using self-reported health status [14], while objective indicators encompass both physical and psychological levels. Physical health is commonly evaluated through one’s ability to perform activities of daily living (ADL) [15] and instrumental ADL (iADL) [16]. Mental health in older adults, specifically depression and cognitive abilities, can be quantified using validated tools such as the Center for Epidemiologic Studies Depression Scale, the Symptom Checklist-90, the Montreal Cognitive Assessment, and the Mini-Mental State Examination (MMSE).

Various factors influence health inequalities among older adults, including residence area (urban or rural) [17,18], socioeconomic status [19], educational attainment [20], race [21], and early life experiences [22]. For instance, men from lower-income groups tend to have higher rates of smoking, leading to a higher prevalence of smoking-related illnesses, thereby contributing to health inequalities among different groups of older adults [23].

### Dimensions of the Digital Divide

The digital divide refers to disparities in accessing, using, and benefiting from digital technologies among various groups. Within the framework of the 3-stage digital divide, this concept can be divided into 3 dimensions: the digital access divide, digital use divide, and digital outcome divide. The accessibility divide arises from limited internet connectivity; lack of computing devices, software, and accessories; and reduced interest in using the internet [24]. Internet access is typically assessed through household devices and network conditions [25,26]. With an increase in internet penetration, the access divide has narrowed, shifting focus toward diversified differences in use patterns and application depth, known as the “internet use divide.” The use divide is related to technical expertise and effective use of digital technologies [27]. Scholars [28,29] have attempted to quantify these differences through use patterns and digital skills to measure individual use variations. The outcome divide dimension refers to differences in offline benefits derived from internet use [26,30]. The weak information acquisition and mastery abilities of older adults are proposed as key reasons for the emergence of the digital divide [31]. Thus, this study focuses on the impact of access and use divides on health inequalities.

### Association Between Health Inequalities and Internet Use

The impact of the digital divide on health inequalities remains contested. Some scholars argue that the spread of the internet has bridged the access divide to some extent, changing health information dissemination patterns and facilitating balanced information flow [32]. This can help reduce health inequalities among older adults, who are often disadvantaged in accessing information [33]. Services derived from digital technology, such as remote consultations, have the potential to alleviate imbalances in health care resource distribution, improving disparities between urban and rural health outcomes [34,35]. However, other studies suggest that while telemedicine can enhance health care access, it may not significantly impact health inequalities [36].

Conversely, it has been argued that the digital divide can exacerbate the inverse care law, potentially excluding those who would benefit most from digitally delivered health care [37]. Research has indicated that older adults may experience negative health effects due to the digital divide hindering their access to mobile health services [38]. A study in the Netherlands found that while older adults benefit from internet use, they do so to a lesser extent than younger people, which further exacerbates health disparities between age groups [39]. Research from Finland suggests that online health care services may intensify existing health disparities due to inequalities in access, skill, and use [40]. In addition, factors such as age [41], sex [42], socioeconomic status [43], and education level [44] influence digital health literacy [45] and telemedicine use, which in turn affect older adults’ ability to understand and use digital health information and services. However, most previous studies exploring the relationship between the digital divide and health inequalities are review articles, with relatively few cross-sectional studies. Moreover, these studies often have limitations such as small sample sizes, unbalanced sex distributions, and higher educational attainment, which may introduce biases

### Objectives

**Figure 1:**
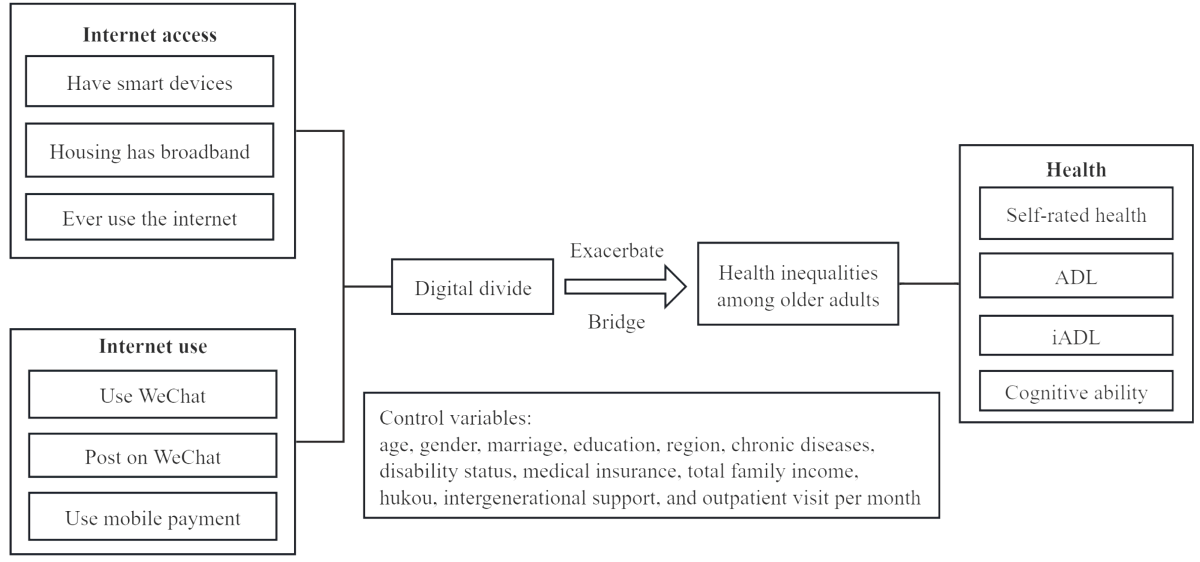
Theoretical framework ("hukou" is a Chinese term for "household registration"). ADL: activity of daily living; iADL: instrumental activity of daily living.

On the basis of the 2018 and 2020 cross-sectional data from the China Health and Retirement Longitudinal Study (CHARLS), this study addresses the limitations of previous research by examining the impact of internet use on health inequality among older adults at a micro level. It focuses on the digital access divide and use divide to investigate their influence on older adults’ health equity, including self-rated health, ADL, iADL [17], and MMSE scores. The theoretical framework is shown in Figure 1. This study is guided by the following research questions: (1) Does access to and use of the internet exacerbate or bridge health disparities among older adults? and (2) Does the impact of internet access and use on health inequality among older adults vary across different demographics?

## Methods

### Data Sources

This study conducted a secondary analysis using data from CHARLS, a nationally representative longitudinal survey conducted in China by Peking University. CHARLS aims to understand the conditions of adults aged ≥45 years, along with their spouses. CHARLS currently possesses 5 waves of data spanning from 2011 to 2020. For this study, data from 2018 and 2020 were used. According to the World Health Organization, older adults are defined as those aged ≥60 years. However, considering that individuals aged 50 to 60 years will soon transition into older adulthood, it is important to study the impact of digital technology on their intentions and behavior; therefore, the age range for this study was ≥50 years. CHARLS 2020 included 19,367 participants; after excluding 2074 (10.71%) participants aged <50 years and 5120 (26.44%) with missing key variable data, 12,173 (62.85%) participants were retained. CHARLS 2018 included 19,827 participants; after excluding 2149 (10.84%) participants aged <50 years and 10,034 (50.61%) with missing key variable data, 7644 (38.55%) participants were retained.

### Ethical Considerations

**Figure 2:**
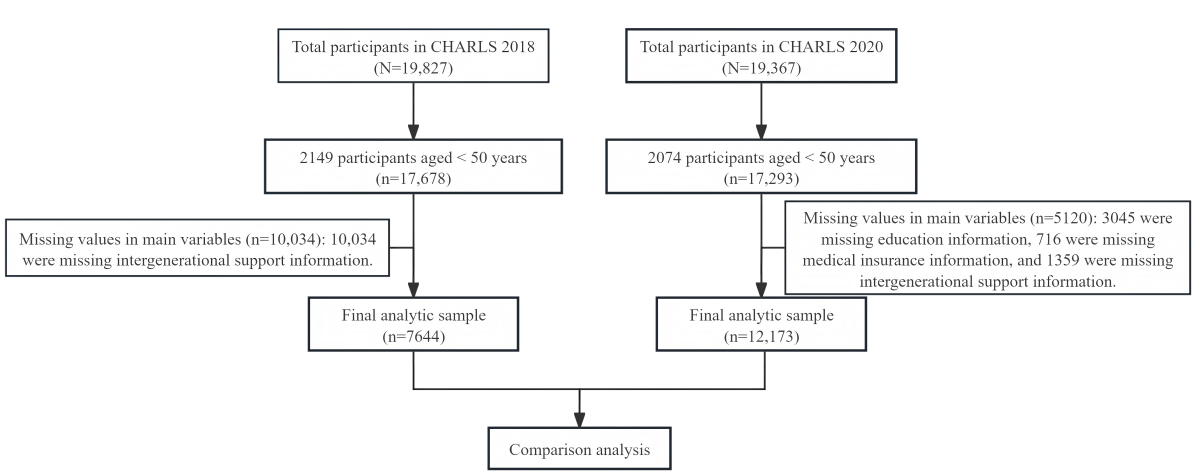
Flowchart of sample selection. CHARLS: China Health and Retirement Longitudinal Study.

Prior the survey, all participants provided written informed consent, and the survey protocols received approval from the Peking University Ethics Review Board (IRB00001052-11015; Figure 2) [46]. The survey was also anonymous, and answers were protected by privacy law.

### Variables and Measurements

#### Dependent Variable

This study used subjective and objective health indicators. Subjective indicators were measured through self-rated health, while objective indicators were divided into physical and psychological dimensions. Physical health was assessed based on the ability to perform ADL and iADL [17], and mental health was evaluated using cognitive function measured by the MMSE. Self-rated health consisted of five levels: (1) very unhealthy, (2) moderately unhealthy, (3) average, (4) moderately healthy, and (5) very healthy.

The ADL scale comprises 6 items: dressing, bathing or showering, self-feeding, getting into or out of bed, using the toilet (including getting up and down), and controlling urination and defecation (including catheter or pouch use). Responses were categorized into 4 levels: no difficulty, difficulty but able to perform, difficulty needing assistance, and unable to perform. Participants indicating *no difficulty* were scored as 1, while those indicating any level of difficulty were scored from 2 to 4 [47,48]. The total score ranges from 6 to 24 points.

The iADL scale includes 6 items: cooking, housework, shopping, managing money, making phone calls, and taking medication. Responses were assessed on a 4-point scale: 1=no difficulty, 2=difficulty but manageable, 3=difficulty needing assistance, and 4=inability to perform. Scores range from 6 to 24 points, with 6 indicating intact daily living skills and a higher score indicating greater impairment [47,48].

Cognitive function was evaluated using the MMSE [49], a concise assessment of adult cognitive status. The MMSE is used for screening cognitive impairment, gauging its severity, monitoring changes over time, and assessing treatment response. It consists of 30 items covering 7 cognitive domains: temporal orientation, spatial orientation, registration, attention and computation, recall, language, and praxis. Scores range from 0 to 30, with higher scores indicating better cognitive function.

According to the relative deprivation theory, older adults with poorer health status experience a greater sense of relative deprivation due to accumulating health disadvantages, leading to increased health inequality. We use the Kakwani relative deprivation index, which combines the results of self-rated health status, ADLs, iADLs, and cognitive function, to measure the degree of health inequality. The Kakwani index satisfies the problems of dimensionlessness, regularity, and transfer invariance. In addition, the Kakwani index can overcome the shortcomings of the Gini coefficient, which does not satisfy the additivity and decomposability. Therefore, this paper uses the Kakwani relative deprivation index to calculate the degree of health inequality of older adults. The measurement method is as follows: *Y* represents the sample size of older adults group of *n*, and arranged in order of the degree of health from smallest to the largest, the health distribution function for this group of older adults is as follows:

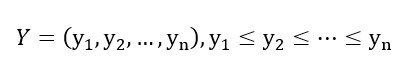

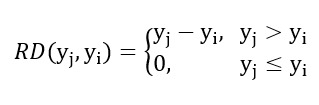





In equation 1, RD (y_j_, y_i_) denotes the relative deprivation index of health of the *j-*th older adult to the *i-*th older adult, and the relative deprivation index of health of the *i-*th older adult *n* is obtained by summing *j* and dividing it by the mean of the health level of all older adults in the group; hence, the degree of health inequality is as follows:

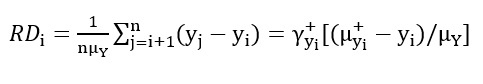



In equation 2, μ_Y_ represents the mean health level of all older adults in Y, 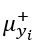 represents the mean health level of samples in Y with a health level of more than y_i_, and 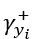 is the number of samples in Y with a health level of more than y_i_ as a proportion of the total sample.

#### Independent Variables

The digital divide is generally considered to have 3 dimensions: access divide, use divide, and outcome divide. This study focused on the first 2 dimensions. Three indicators were used to measure internet access level: the number of smart devices owned, broadband access at home, and the number of digital devices owned. The indicators were converted into a binary variable, where values 1 to 2 were assigned as 1. If any of the 3 indicators for internet access were 1, the individual was considered to have internet access.

To measure internet use level, 3 indicators were chosen: use of WeChat perform for socializing, posting on WeChat Moments, and using mobile payments [27]. WeChat, owned by the Chinese tech giant Tencent, is comparable to other social networking sites (SNSs) in western countries, such as Facebook and Twitter. The results of each question were denoted as 0 or 1, with higher values representing greater internet use capacity (ranging from 0 to 3).

#### Control Variables

Control variables were organized based on demographic characteristics: sex [50] (male=1 and female=0), age [51] (continuous variable), marital status [52] (married=1 and unmarried=0, where “unmarried” includes separation, divorce, widowhood, and never married), education [53] (no formal education=0, elementary school=1, and junior high school and above=2), chronic diseases [54] (yes=1 and no=0), disability status [55] (yes=1 and no=0), medical insurance [56] (urban employee medical insurance=1, urban and rural resident medical insurance=2, and other medical insurance=3), total family income [57] (log-transformed after bilateral trimming), *hukou* [58] (household registration; urban=0 and rural=1), intergenerational support [59] (determined by frequency of interactions with children; yes=1 and no=0), and outpatient visits [4] per month (range 0-30). Region of residence [60] (east, central, west, and northwest) was represented by 3 dummy variables.

### Statistical Methods

#### Overview

Statistical analyses were conducted using Stata (version 15.0; StataCorp). Statistical significance was defined as 2-tailed *P* values <.05. Continuous data were presented as means and SDs, while categorical data were expressed as frequencies and percentages. The relationship between digital access (use) and health inequalities was examined using univariate analysis. Categorical variables were assessed using the chi-square test, continuous variables following a normal distribution were analyzed using the 2-tailed *t* test, and continuous variables not conforming to a normal distribution were evaluated using the Wilcoxon rank sum test.

#### Multiple Linear Regression

Multiple linear regression models were used to test the association between multiple dimensions of the digital divide and health inequalities. Subgroup analyses were conducted based on sex, age, *hukou* (household registration), educational level, and region. In addition to fully adjusted models, moderated effect analyses were conducted using interaction terms of internet access and use with other covariates to determine if the association between the digital divide and health inequality was moderated by these factors.

#### Propensity Score Matching Method

Sensitivity analyses were performed using propensity score matching (PSM). To analyze the impact of internet access and use on health inequality among older adults, the sample was divided into an experimental group (those who accessed or used the internet) and a control group (those who did not access or use the internet). Internet access (binary categorical variable) and internet use (converted into a binary variable using the median) were core variables for matching, and the nearest neighbor matching (1:1) principle was used to calculate the propensity score.

To further analyze the effects and mechanisms of internet access and use on health inequalities among older adults, the following model was constructed:

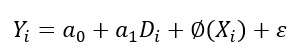



Where Y*i* denotes the degree of health inequality among older adults, D_i_ denotes the access or use of the Internet among older adults, X_i_ denotes other influences on the degree of health inequality among older adults, and ε is a random perturbation term. On the basis of the PSM model, internet access and internet use is set as a binary variable, and D_i_ = {0,1} denotes whether the *i-*th older adult has access or use of the internet or not, that is, D_i_=1 is access or use of the internet, and D_i_=0 is no access or no use of the internet. There are 2 different scenarios of health inequality among older adults, one is the level of health inequality among older adults when accessing or using the internet (y_1i_) and the other is the level of health inequality among older adults when not accessing or not using the internet (y_0i_). The average treatment effect for the impact of the internet on health inequalities was calculated as:

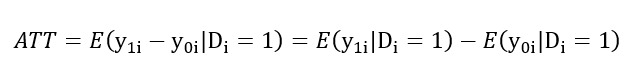



In equation 5, average treatment effect denotes the difference between the level of health inequality among older adults with access and use of the internet E(y_1i_|Di=1) and the level of health inequality among older adults without access and use of the internet E(y_0i_|Di=1). Due to the unavailability of E(y_0i_|Di=1), E(y_0i_|Di=0) for the control group was used in the model as a proxy for the level of health inequality among older adults who use the internet.

## Results

### Characteristics of Study Participants

Table 1 presents the descriptive statistics of the sample, comprising 7644 observations from 2018 and 12,173 observations from 2020. In 2018, in total, 48.59% (3714/7644) of the participants were male, while 51.41% (3930/7644) were female. The average age of older adults was 65.11 (SD 10.02) years. In total, 73.31% (5604/7644) of the participants were married at the time of the survey. Given that the average age of participants was approximately 65 years and education levels in China were generally low in the 1960s, this study classified participants with an education level of junior high school or above as “better educated.” The descriptive results indicate that only 30.65% (2343/7644) of the participants met this criterion. Most participants were rural residents (6449/7644, 84.37%). More than half of the respondents (4095/7644, 53.57%) had chronic diseases, and 14.21% (1086/7644) of the respondents had disabilities. Furthermore, 62.17% (4752/7644) of the respondents reported strong intergenerational support, indicating a tendency to contact and communicate with their children. Geographically, the distribution of older adults was fairly even across the eastern, central, and western regions, with the western region having the highest percentage (2642/7644, 34.56%) and the northeastern region having the fewest (491/7644, 6.43%).

**Table 1:** Characteristics of the study participants.

Variable			Total (N=19,817)		2018 (n=7644)		2020 (n=12,173)
**Broadband access, n (%)**							
	No	9845 (49.68)		4646 (60.78)		5199 (42.71)	
	Yes	9972 (50.32)		2998 (39.22)		6974 (57.29)	
**Number of smart devices owned, n (%)**							
	0	14,693 (74.14)		6841 (89.50)		7852 (64.50)	
	1	4570 (23.06)		651 (8.52)		3919 (32.20)	
	≥2	554 (2.80)		152 (1.98)		402 (3.30)	
**Mobile payment use, n (%)**							
	No	2230 (11.25)		387 (5.06)		1843 (15.14)	
	Yes	2894 (14.61)		416 (5.44)		2478 (20.36)	
	Unknown	14,693 (74.14)		6841 (89.50)		7852 (64.50)	
**WeChat use, n (%)**							
	No	512 (2.59)		54 (0.70)		458 (3.76)	
	Yes	4612 (23.27)		749 (9.80)		3863 (31.74)	
	Unknown	14,693 (74.14)		6841 (89.50)		7852 (64.50)	
**WeChat Moments use, n (%)**							
	No	1870 (9.43)		199 (2.60)		1671 (13.72)	
	Yes	2742 (13.84)		550 (7.20)		2192 (18.01)	
	Unknown	15,205 (76.73)		6895 (90.20)		8310 (68.27)	
Age (y), mean (SD)			64.41 (9.54)		65.11 (10.02)		63.97 (9.20)
**Sex** **, n (%)**							
	Female	10,201 (51.48)		3930 (51.41)		6271 (51.52)	
	Male	9616 (48.52)		3714 (48.59)		5902 (48.48)	
**Marriage, n (%)**							
	Married	15,465 (78.04)		5604 (73.31)		9861 (81.01)	
	Unmarried	4352 (21.96)		2040 (26.69)		2312 (18.99)	
**Education, n (%)**							
	No formal education	4810 (24.27)		1917 (25.08)		2893 (23.77)	
	Elementary school	8610 (43.45)		3384 (44.27)		5226 (42.93)	
	Junior high school and above	6397 (32.28)		2343 (30.65)		4054 (33.30)	
**Current address, n (%)**							
	Rural	15,905 (80.26)		6449 (84.37)		9456 (77.68)	
	Urban	3912 (19.74)		1195 (15.63)		2717 (22.32)	
**Medical insurance, n (%)**							
	Urban employee medical insurance	2414 (12.33)		759 (10.25)		1655 (13.60)	
	Urban and rural resident medical insurance	11,058 (56.49)		849 (11.47)		10,209 (83.87)	
	Other medical insurance	6104 (31.18)		5795 (78.28)		309 (2.53)	
**Chronic disease, n (%)**							
	No	11,361 (57.33)		4095 (53.57)		7266 (59.69)	
	Yes	8456 (42.67)		3549 (46.43)		4907 (40.31)	
**Disability, n (%)**							
	No	16,943 (85.50)		6558 (85.79)		10,385 (85.31)	
	Yes	2874 (14.50)		1086 (14.21)		1788 (14.69)	
**Intergenerational support, n (%)**							
	Weak	9775 (49.33)		2892 (37.83)		6883 (56.54)	
	Strong	10.042 (50.67)		4752 (62.17)		5290 (43.46)	
**Region, n (%)**							
	Eastern region	5859 (29.57)		2089 (27.33)		3770 (30.96)	
	Central region	6105 (30.80)		2422 (31.68)		3683 (30.26)	
	Western region	6649 (33.55)		2642 (34.56)		4007 (32.92)	
	Northeast region	1204 (6.08)		491 (6.43)		713 (5.86)	
Log(income), mean (SD)			0.72 (0.34)		0.71 (0.35)		0.72 (0.33)
Outpatient visit per month, mean (SD)			0.45 (1.43)		0.39 (1.37)		0.49 (1.47)
**Health, n (%)**							
	Very good	1718 (8.67)		500 (6.54)		1218 (10.01)	
	Good	3072 (15.50)		1737 (22.72)		1335 (11.00)	
	Fair	8992 (45.38)		3431 (44.89)		5561 (45.68)	
	Poor	3107 (15.68)		874 (11.43)		2233 (18.34)	
	Very poor	1572 (7.93)		769 (10.06)		803 (6.60)	
	Unknown	1356 (6.84)		333 (4.36)		1023 (8.37)	
ADL^a^, mean (SD)			6.43 (3.44)		5.93 (4.79)		6.75 (2.15)
iADL, mean (SD)			7.88 (4.14)		8.82 (5.15)		7.29 (3.22)
Cognition, mean (SD)			20.76 (8.3)		22.90 (7.97)		19.53 (8.04)

^a^ADL: Activities Of Daily Living scale.

^b^iADL: Instrumental Activities of Daily Living scale.

In 2020, in total, 48.48% (5902/12,173) of the participants were male and 51.52% (6271/12,173) were female. The mean age of older adults was 63.97 (SD 9.20) years. More than 80% (9861/12,173) of the participants were married. For educational attainment, only 33.3% (4054/12,173) of the participants had a junior high school education or higher. Most older adults were rural residents (9456/12,173, 77.68%), and the primary health insurance was urban and rural residents’ health insurance (10,209/12,173, 83.87%). More than half of the participants (7266/12,173, 59.69%) had chronic diseases, while 14.69% (1788/12,173) had disabilities. In addition, 43.46% (5290/12,173) of the participants reported strong intergenerational support. The regional distribution of older adults in 2020 was similar to that in 2018, with the Western region having the highest proportion (4007/12,173, 32.92%) and the Northeast having the lowest (713/12,173, 5.86%).

Regarding internet access and use among older adults, only 39.22% (2998/7644) of the households had broadband in 2018, whereas by 2020, more than half of the households (6974/12,173, 57.29%) had broadband. Smart device ownership also increased significantly, with only 8.52% (651/7644) of the older adults owning a smartphone in 2018 compared to 32.2% (3919/12,173) in 2020.

The health status of older adults was also analyzed descriptively. For subjective health evaluations, the largest proportion of participants in both 2018 and 2020 rated their health as “fair” (3431/7644, 44.89% and 5561/12,173, 45.68%, respectively). However, the proportion of participants rating their health as “very good” increased in 2020 compared to 2018, accounting for 10.01% (1218/12,173). In terms of objective health indicators, older adults had better ADL scores in 2018 compared to 2020, while iADL scores were worse in 2018 compared to 2020. Finally, older adults had higher cognitive ability scores in 2018, with an average score of 22.90 (SD 7.97), compared to 2020.

### Results of Simple Regression

This section investigated the effects of internet access and use on health inequalities among older adults, using data from the CHARLS collected in 2018 and 2020. In CHARLS 2018, the findings indicated that internet access and use generally widened health inequalities among older adults. Specifically, internet access was found to have amplified disparities in subjective self-rated health, ADL, and cognitive ability, with effect sizes of 3.9%, 5.8%, and 7.5%, respectively, all of which were statistically significant. In contrast, internet access showed a significant narrowing effect on inequalities related to iADL, with an effect size of 5.1%. Similarly, internet use was associated with an increase in inequalities in subjective self-rated health and cognitive ability, with effect sizes of 7.5% and 7.6%, respectively, and these results were statistically significant. However, internet use had a mitigating effect on iADL and ADL inequalities, with effect values of 5% and 0.8%, although these effects were not statistically significant (Tables 2 and 3).

**Table 2:** Regression results for health based on internet access level in 2018 (N=7644)^a,b^.

Attribute and level	Self-rated health^c^			ADL^d^			iADL^e^			Cognition^f^	
	β coefficient (SE)	*P* value	β coefficient (SE)		*P* value	β coefficient (SE)		*P* value	β coefficient (SE)		*P* value
Internet access level	0.039 (0.005)	.001	0.058 (0.008)		<.001	–0.051 (0.003)		<.001	0.075 (0.008)		<0.001
Male	0.088 (0.005)	<.001	0.194 (0.008)		<.001	0.095 (0.003)		<.001	–0.086 (0.008)		<.001
Age	–0.075 (0.0003)	<.001	–0.222 (0.0004)		<.001	–0.311 (0.0001)		<.001	–0.504 (0.001)		<.001
Rural	–0.033 (0.006)	.006	–0.007 (0.012)		.57	0.039 (0.003)		<.001	0.037 (0.010)		<.001
Secondary schools	–0.052 (0.006)	<.001	0.013 (0.009)		.32	0.142 (0.004)		<.001	–0.212 (0.010)		<.001
Junior high school and above	0.012 (0.007)	.49	0.095 (0.012)		<.001	0.130 (0.004)		<.001	–0.178 (0.012)		<.001
Married	–0.002 (0.006)	.87	–0.005 (0.009)		.63	0.047 (0.003)		<.001	–0.155 (0.009)		<.001
Urban and rural resident medical insurance	0.006 (0.007)	.60	0.002 (0.012)		.85	0.017 (0.004)		.10	–0.021 (0.011)		.03
Chronic	–0.174 (0.004)	<.001	–0.126 (0.008)		<.001	–0.035 (0.002)		.001	–0.014 (0.007)		.16
Disability	–0.096 (0.005)	<.001	–0.086 (0.010)		<.001	–0.074 (0.004)		<.001	0.029 (0.011)		.009
Outpatient visit	0.085 (0.002)	<.001	–0.071 (0.003)		<0.001	–0.039 (0.001)		.001	0.005 (0.003)		.643
Intergenerational support	–0.031 (0.005)	.006	0.020 (0.008)		.06	0.072 (0.003)		<.001	–0.051 (0.008)		<.001
Log(income)	0.015 (0.006)	.19	0.021 (0.011)		.05	0.052 (0.003)		<.001	–0.013 (0.010)		.20
Central region	–0.047 (0.006)	.001	–0.041 (0.010)		.002	0.086 (0.003)		<.001	–0.009 (0.009)		.45
Western region	–0.055 (0.006)	<.001	–0.040 (0.010)		.002	0.104 (0.003)		<.001	–0.011 (0.009)		39
Northeast region	0.006 (0.010)	.64	–0.039 (0.017)		.001	–0.020 (0.005)		.07	–0.004 (0.015)		.69

^a^*R*^2^ for the above models: 0.0901, 0.1779, 0.2138, and 0.2482.

^b^Perform 2-tailed winsorization and logarithmic transformation on income.

^c^Self-rated health (reference: reporting negative self-reported health).

^d^ADL: activity of daily living. Reporting difficulty with ADL (reference: no).

^e^iADL: instrumental activity of daily living. Reporting difficulty with iADL (reference: no).

^f^Cognition (reference: reporting negative cognition).

**Table 3:** Regression results for health based on internet use level in 2018 (N=1187)^a^.

Attribute and level	Self-rated health			ADL^b^			iADL^c^			Cognition	
	β coefficient (SE)	*P* value	β coefficient (SE)		*P* value	β coefficient (SE)		*P* value	β coefficient (SE)		*P* value
Internet use level	0.075 (0.008)	.03	0.076 (0.016)		.03	–0.050 (0.004)		.16	–0.008 (0.010)		.77
Male	0.086 (0.015)	02	0.206 (0.028)		<.001	0.064 (0.006)		.06	–0.029 (0.015)		.20
Age	–0.116 (0.001)	.002	–0.194 (0.002)		<.001	–0.202 (0.001)		<.001	–0.772 (0.002)		<.001
Rural	–0.073 (0.016)	.045	0.010 (0.030)		.78	0.199 (0.007)		<.001	–0.012 (0.017)		.62
Secondary schools	0.023 (0.053)	.84	0.025 (0.091)		.80	0.248 (0.025)		.04	–0.214 (0.058)		.007
Junior high school and above	0.037 (0.053)	.74	0.162 (0.091)		.11	0.273 (0.025)		.03	–0.210^*^ (0.058)		.01
Married	0.052 (0.021)	.11	–0.043 (0.042)		.20	0.006 (0.009)		.86	–0.050 (0.027)		.06
Urban and rural resident medical insurance	–0.023 (0.028)	.53	0.026 (0.047)		.41	0.013 (0.011)		.70	–0.001 (0.024)		.97
Chronic	–0.123 (0.015)	.001	–0.094 (0.029)		.008	–0.017 (0.006)		.60	–0.005 (0.015)		.82
Disability	–0.085 (0.024)	.007	–0.110 (0.043)		<.001	0.013 (0.011)		.72	0.012 (0.026)		.60
Outpatient visit	–0.114 (0.006)	.001	–0.080 (0.010)		.01	–0.081 (0.003)		.03	–0.040 (0.007)		.15
Intergenerational support	0.027 (0.019)	.42	0.005 (0.036)		.89	0.027 (0.007)		.36	0.026 (0.019)		.24
Log(income)	0.006 (0.024)	.88	0.047 (0.041)		.18	0.079 (0.009)		.02	–0.038 (0.022)		.11
Central region	0.031 (0.019)	.47	–0.059 (0.035)		.14	0.251 (0.007)		<.001	0.001 (0.018)		.97
Western region	–0.019 (0.020)	.65	–0.083 (0.037)		.04	0.217 (0.008)		<.001	–0.002 (0.021)		.96
Northeast region	0.016 (0.026)	.68	–0.040 (0.047)		.28	0.207 (0.011)		.48	0.028 (0.026)		.28

^a^*R*^2^ for the above models: 0.0928, 0.1640, 0.1952, and 0.6065.

^b^ADL: activity of daily living.

^c^iADL: instrumental activity of daily living.

Interestingly, the findings for CHARLS 2020 revealed a contrasting trend. In 2020, internet access was associated with a reduction in health inequalities in subjective self-rated health, ADL, iADL, and cognitive ability, with effect values of 3.8%, 2.1%, 3.5%, and 7.5%, respectively, all of which were statistically significant except for ADL. Likewise, internet use exhibited a narrowing effect on health inequalities, with respective effect sizes for subjective self-rated health, ADL, iADL, and cognition being 4.8%, 0.3%, 1.7%, and 12.8%. Except for ADL and iADL, these results were statistically significant. Notably, internet access and use demonstrated the largest impact on cognitive inequalities, highlighting cognition as a critical area affected by the digital divide (Tables 4 and 5).

**Table 4:** Regression results for health based on internet access level in 2020 (N=12,173)^a^.

Attribute and level	Self-rated health			ADL^b^			iADL^c^			Cognition	
	β coefficient (SE)	*P* value	β coefficient (SE)		*P* value	β coefficient (SE)		*P* value	β coefficient (SE)		*P* value
Internet access level	–0.038 (0.004)	<.001	–0.021 (0.001)		.053	–0.035 (0.001)		<.001	–0.075 (0.005)		<.001
Male	–0.054 (0.003)	<.001	0.059 (0.001)		<.001	0.058 (0.001)		<.001	–0.005 (0.004)		.56
Age	–0.009 (0.0002)	.38	–0.082 (0.0001)		.001	–0.129 (0.0001)		<.001	0.155 (0.0003)		<.001
Rural	0.022 (0.004)	.02	0.010 (0.001)		.28	0.011 (0.001)		.24	0.031 (0.005)		<.001
Secondary schools	0.007 (0.005)	.59	–0.005 (0.001)		.65	0.042 (0.002)		<.001	–0.372 (0.007)		<.001
Junior high school and above	–0.026 (0.005)	.06	0.022 (0.001)		.08	0.077 (0.002)		<.001	–0.483 (0.007)		<.001
Married	0.016 (0.005)	.11	–0.022 (0.001)		.04	–0.022 (0.002)		.02	–0.057 (0.006)		<.001
Urban and rural resident medical insurance	0.038 (0.004)	<.001	0.053 (0.001)		<.001	0.042 (0.002)		<.001	0.041 (0.005)		<.001
Chronic	0.156 (0.003)	<.001	–0.044 (0.001)		<.001	–0.047 (0.001)		<.001	0.012 (0.004)		.13
Disability	0.271 (0.006)	<.001	–0.227 (0.001)		<.001	–0.337 (0.002)		<.001	0.092 (0.008)		<.001
Outpatient visit	0.155 (0.002)	<.001	–0.043 (0.0003)		<.001	–0.037 (0.0004)		<.001	–0.019 (0.001)		.02
Intergenerational support	–0.011 (0.003)	.24	–0.009 (0.001)		.31	0.005 (0.001)		.57	–0.024 (0.004)		.003
Log(income)	0.009 (0.005)	.32	0.036 (0.001)		<.001	0.050 (0.002)		<.001	–0.094 (0.007)		<.001
Central region	0.005 (0.004)	.59	0.102 (0.001)		<.001	0.110^***^ (0.001)		<.001	0.030 (0.005)		.002
Western region	0.013 (0.004)	.21	0.070 (0.001)		<.001	0.066 (0.001)		<.001	0.008 (0.005)		.43
Northeast region	0.024 (0.007)	.005	0.076 (0.002)		<.001	0.093 (0.002)		<.001	–0.003 (0.008)		.67

^a^*R*^2^ for the above models: 0.1639, 0.0972, 0.1973, and 0.3058.

^b^ADL: activity of daily living.

^c^iADL: instrumental activity of daily living.

**Table 5:** Regression results for health based on internet use level (N=4321) in 2020^a^.

Attribute and level	Self-rated health			ADL^b^			iADL^c^			Cognition	
	β coefficient (SE)	*P* value	β coefficient (SE)		*P* value	β coefficient (SE)		*P* value	β coefficient (SE)		*P* value
Internet use level	–0.048 (0.003)	.003	–0.003 (0.001)		.85	–0.017 (0.001)		.27	–0.128 (0.003)		<.001
Male	–0.051 (0.005)	.001	0.081 (0.001)		<.001	0.068 (0.002)		<.001	0.025 (0.005)		.10
Age	0.010 (0.0004)	.53	–0.106 (0.0001)		<.001	–0.100 (0.0001)		<.001	0.044^*^ (0.0004)		.01
Rural	0.015 (0.005)	.37	0.031 (0.001)		.06	0.029 (0.002)		.08	–0.011 (0.005)		.497
Secondary schools	–0.004 (0.012)	.90	–0.017 (0.002)		.58	0.038 (0.004)		.27	–0.373 (0.015)		<.001
Junior high school and above	–0.030 (0.012)	.42	0.014 (0.002)		.66	0.095 (0.004)		.008	–0.531 (0.015)		<.001
Married	–0.023 (0.008)	.15	–0.009 (0.002)		.55	–0.029 (0.003)		.06	–0.050 (0.009)		.005
Urban and rural resident medical insurance	0.051 (0.006)	.001	0.105 (0.001)		<.001	0.095 (0.002)		<.001	0.038 (0.006)		.02
Chronic	0.164 (0.005)	<.001	–0.029 (0.001)		.049	–0.033 (0.002)		.03	0.047 (0.005)		.002
Disability	0.247 (0.015)	<.001	–0.138 (0.003)		<.001	–0.225 (0.004)		<.001	0.058 (0.013)		.003
Outpatient visit	0.176 (0.002)	<.001	–0.038 (0.001)		.04	–0.053 (0.001)		.008	–0.029 (0.001)		.005
Intergenerational support	–0.023 (0.005)	.11	0.019 (0.001)		.20	0.007 (0.002)		.65	–0.022 (0.005)		.13
Log(income)	–0.018 (0.007)	.23	0.047 (0.002)		.001	0.056 (0.002)		<.001	–0.051 (0.008)		.003
Central region	0.018 (0.006)	.28	0.176 (0.001)		<.001	0.168 (0.002)		<.001	0.046 (0.006)		.008
Western region	0.039 (0.006)	.02	0.116 (0.001)		<.001	0.127 (0.002)		<.001	0.001 (0.006)		.95
Northeast region	0.023 (0.008)	.10	0.143 (0.002)		<.001	0.172 (0.003)		<.001	0.015 (0.009)		.37

^a^*R*^2^ for the above models: 0.1752, 0.0941, 0.1336, and 0.1397.

^b^ADL: activity of daily living.

^c^iADL: instrumental activity of daily living.

### Sensitivity Analysis: PSM

To assess the robustness of the results, PSM was performed to estimate the net effect of internet access and use on health inequalities among older adults. Before applying PSM, a balance test was conducted to ensure comparability between the treatment and control groups after matching, apart from the independent variables. The kernel density trend graphs for the 2 subsamples indicated improved convergence after matching across different submodels, with satisfactory results for sample support and equilibrium tests (Multimedia Appendix 1). In addition, standardized mean differences were used to measure the balance of covariates before and after matching. Table S1 in Multimedia Appendix 1 shows that the standardized mean differences for all key variables were reduced to <20% after matching, indicating adequate balance and supporting the robustness of the procedure.

The results of the PSM analysis were consistent with the regression analysis. In 2018, internet access significantly widened health inequalities, particularly in subjective self-rated health, ADL, and cognition, with effect sizes of 2%, 6.5%, and 2.2%, respectively. Internet use also widened disparities in subjective health and ADL, with effect sizes of 5.7% and 8.4%, respectively. However, in 2020, internet access and use showed a trend toward reducing health inequalities, with the greatest effect seen in narrowing cognitive disparities, at 4.6% and 5.2%, respectively. Overall, the consistency between the regression and PSM results affirms the robustness of the model (Tables 6 and 7).

**Table 6:** Sensitivity analysis using propensity score matching for the year 2018.

Attribute and levels	Internet access level					Internet use level			
	Treatment group	Control group	ATT^a^ (*P* value)	SE	Treatment group		Control group	ATT (*P* value)	SE
Self-rated health	0.192	0.173	0.020 (.008)	0.008	0.241		0.184	0.057 (.001)	0.024
ADL^b^	0.454	0.389	0.065 (<.001)	0.013	0.556		0.472	0.084 (.03)	0.043
iADL^c^	0.243	0.248	–0.005 (.26)	0.004	0.260		0.261	–0.001 (.55)	0.010
Cognition	0.432	0.410	0.022 (.01)	0.013	0.495		0.507	–0.012 (.13)	0.038

^a^ATT: average treatment effect.

^b^ADL: activity of daily living.

^c^iADL: instrumental activity of daily living.

**Table 7:** Sensitivity analysis using propensity score matching for the year 2020.

Attribute and levels	Internet access level					Internet use level			
	Treatment group	Control group	ATT^a^ (*P* value)	SE	Treatment group		Control group	ATT (*P* value)	SE
Self-rated health	0.166	0.185	–0.020 (<.001)	0.008	0.143		0.167	–0.025 (<.001)	0.009
ADL^b^	0.099	0.101	–0.00 (.68)	0.002	0.102		0.103	–0.001 (.47)	0.002
iADL^c^	0.159	0.162	–0.003 (.74)	0.003	0.167		0.173	–0.005 (.04)	0.003
Cognition	0.173	0.219	–0.046 (<.001)	0.011	0.092		0.144	–0.052 (<.001)	0.010

^a^ATT: average treatment effect.

^b^ADL: activity of daily living.

^c^iADL: instrumental activity of daily living.

### Heterogeneity Analysis

To further explore the effect of internet access and use on health inequalities, a heterogeneity analysis was conducted using the 2020 CHARLS data, focusing on sex, age, education level, and urban and rural status. The subgroup analysis demonstrated that internet access had a stronger impact on reducing health inequalities among rural residents compared to urban residents. For rural populations, greater internet access significantly contributed to reducing health disparities (Table 8). Sex differences showed that internet use had a larger impact on reducing inequalities among males, with an effect value of 9.5% (*P*<.001). Regarding age, younger adults aged <60 years experienced greater benefits from internet access in reducing inequalities in self-assessed health and cognitive ability, with effect values of 5.6% (*P*<.01) and 14.8% (*P*<.001), respectively. Regarding education level, both internet access and use had a significant impact on reducing health inequalities among those with higher education, potentially due to their greater information literacy and ability to access health services online. Complete results are shown in Tables 8-15.

**Table 8:** Results for urban and rural subgroups at the internet access level^a^.

Attribute and levels	Urban subgroups (n=2717)				Rural subgroups (n=9456)			
	Self-rated health, β coefficient (*P* value)	ADL^b^, β coefficient (*P* value)	iADL^c^, β coefficient (*P* value)	Cognition, β coefficient (*P* value)	Self-rated health, β coefficient (*P* value)	ADL, β coefficient (*P* value)	iADL, β coefficient (*P* value)	Cognition, β coefficient (*P* value)
Internet access level	–0.042 (.06)	–0.016 (.48)	–0.036 (.10)	–0.055 (.01)	–0.036 (.001)	–0.022 (.07)	–0.036 (.001)	–0.076 (<.001)
Control	Yes	Yes	Yes	Yes	Yes	Yes	Yes	Yes
*R* ^2^	0.191	0.118	0.218	0.240	0.156	0.093	0.196	0.302

^a^The numbers in the table represent the standardized β coefficients for each submodel.

^b^ADL: activity of daily living.

^c^iADL: instrumental activity of daily living.

**Table 9:** Results for urban and rural subgroups at the internet use level^a^.

Attribute and levels	Urban subgroups (n=1524)					Rural subgroups (n=2797)			
	Self-rated health, β coefficient (*P* value)	ADL^b^, β coefficient (*P* value)	iADL^c^, β coefficient (*P* value)	Cognition, β coefficient (*P* value)	Self-rated health, β coefficient (*P* value)		ADL, β coefficient (*P* value)	iADL, β coefficient (*P* value)	Cognition, β coefficient (*P* value)
Internet use level	–0.030 (.29)	–0.043 (.11)	–0.019 (.48)	–0.175 (<.001)	–0.056 (.004)		0.015(.46)	–0.020 (.29)	–0.109 (<.001)
Control	Yes	Yes	Yes	Yes	Yes		Yes	Yes	Yes
*R* ^2^	0.199	0.125	0.150	0.103	0.165		0.078	0.134	0.159

^a^The numbers in the table represent the standardized regression coefficients for each submodel.

^b^ADL: activity of daily living.

^c^iADL: instrumental activity of daily living.

**Table 10:** Results for sex subgroups at the internet access level^a^.

Attribute and levels	Female (n=6271)				Male (n=5902)			
	Self-rated health, β coefficient (*P* value)	ADL^b^, β coefficient (*P* value)	iADL^c^, β coefficient (*P* value)	Cognition, β coefficient (*P* value)	Self-rated health, β coefficient (*P* value)	ADL, β coefficient (*P* value)	iADL, β coefficient (*P* value)	Cognition, β coefficient (*P* value)
Internet access level	–0.038 (.006)	–0.031 (.04)	–0.046 (.001)	–0.073 (<.001)	–0.036 (.01)	–0.011 (.47)	–0.023 (.09)	–0.079 (<.001)
Control	Yes	Yes	Yes	Yes	Yes	Yes	Yes	Yes
*R* ^2^	0.155	0.094	0.189	0.345	0.166	0.097	0.199	0.222

^a^The numbers in the table represent the standardized regression coefficients for each submodel.

^b^ADL: activity of daily living.

^c^iADL: instrumental activity of daily living.

**Table 11:** Results for sex subgroups at the internet use level^a^.

Attribute and levels	Female (n=2019)					Male (n=2302)			
	Self-rated health, β coefficient (*P* value)	ADL^b^, β coefficient (*P* value)	iADL^c^, β coefficient (*P* value)	Cognition, β coefficient (*P* value)	Self-rated health, β coefficient (*P* value)		ADL, β coefficient (*P* value)	iADL, β coefficient (*P* value)	Cognition, β coefficient (*P* value)
Internet use level	0.002 (.94)	–0.026 (.29)	–0.030 (.22)	–0.135 (<.001)	–0.095 (<.001)		0.012 (.59)	–0.008 (.70)	–0.124 (<.001)
Control	Yes	Yes	Yes	Yes	Yes		Yes	Yes	Yes
*R* ^2^	0.181	0.102	0.125	0.183	0.168		0.082	0.137	0.097

^a^The numbers in the table represent the standardized regression coefficients for each submodel.

^b^ADL: activity of daily living.

^c^iADL: instrumental activity of daily living.

**Table 12:** Results for age subgroups at the internet access level.

Attribute and levels	<60 years (n=4337)					≥60 years (n=7836)			
	Self-rated health, β coefficient (*P* value)	ADL^b^, β coefficient (*P* value)	iADL^c^, β coefficient (*P* value)	Cognition, β coefficient (*P* value)	Self-rated health, β coefficient (*P* value)		ADL, β coefficient (*P* value)	iADL, β coefficient (*P* value)	Cognition, β coefficient (*P* value)
Internet access level	–0.056 (.001)	–0.007 (.70)	0.003 (.88)	–0.148 (<.001)	–0.027 (.03)		–0.026 (.04)	–0.043 (<.001)	–0.050 (<.001)
Control	Yes	Yes	Yes	Yes	Yes		Yes	Yes	Yes
*R* ^2^	0.178	0.043	0.098	0.201	0.158		0.111	0.213	0.295

^a^The numbers in the table represent the standardized regression coefficients for each submodel.

^b^ADL: activity of daily living.

^c^iADL: instrumental activity of daily living.

**Table 13:** Results for age subgroups at the internet use level^a^.

Attribute and levels	<60 years (n=2649)				≥60 years (n=1672)			
	Self-rated health, β coefficient (*P* value)	ADL^b^, β coefficient (*P* value)	iADL^c^, β coefficient (*P* value)	Cognition, β coefficient (*P* value)	Self-rated health, β coefficient (*P* value)	ADL, β coefficient (*P* value)	iADL, β coefficient (*P* value)	Cognition, β coefficient (*P* value)
Internet use level	–0.047 (.03)	–0.019 (.34)	–0.049 (.01)	–0.115 (<.001)	–0.051 (.04)	0.025 (.32)	0.026 (.30)	–0.139 (<.001)
Control	Yes	Yes	Yes	Yes	Yes	Yes	Yes	Yes
*R* ^2^	0.181	0.067	0.114	0.125	0.174	0.129	0.156	0.156

^a^The numbers in the table represent the standardized regression coefficients for each submodel.

^b^ADL: activity of daily living.

^c^iADL: instrumental activity of daily living.

**Table 14:** Results for education subgroups at the internet access level^a^.

Attribute and levels	No formal education (n=2893)					Elementary school (n=5226)					Junior high school and above (n=4054)			
	Self-rated health, β coefficient (*P* value)	ADL^b^, β coefficient (*P* value)	iADL^c^, β coefficient (*P* value)	Cognition, β coefficient (*P* value)	Self-rated health, β coefficient (*P* value)		ADL, β coefficient (*P* value)	iADL, β coefficient (*P* value)	Cognition, β coefficient (*P* value)	Self-rated health, β coefficient (*P* value)		ADL^b^, β coefficient (*P* value)	iADL^c^, β coefficient (*P* value)	Cognition, β coefficient (*P* value)
Internet access level	–0.004 (.83)	–0.040 (.04)	–0.049 (.006)	–0.074 (<.001)	–0.039 (.007)		0.002 (.92)	–0.005 (.73)	–0.101 (<.001)	–0.060 (.001)		–0.026 (.19)	–0.047 (.01)	–0.048 (.02)
Control	Yes	Yes	Yes	Yes	Yes		Yes	Yes	Yes	Yes		Yes	Yes	Yes
*R* ^2^	0.132	0.111	0.222	0.137	0.161		0.089	0.170	0.102	0.182		0.094	0.160	0.049

^a^The numbers in the table represent the standardized regression coefficients for each submodel.

^b^ADL: activity of daily living.

^c^iADL: instrumental activity of daily living.

**Table 15:** Results for education subgroups at the internet use level^a^.

Attribute and levels	No formal education (n=279)					Elementary school (n=1517)					Junior high school and above (n=2525)			
	Self-rated health, β coefficient (*P* value)	ADL^b^, β coefficient (*P* value)	iADL^c^, β coefficient (*P* value)	Cognition, β coefficient (*P* value)	Self-rated health, β coefficient (*P* value)		ADL^b^, β coefficient (*P* value)	iADL^c^, β coefficient (*P* value)	Cognition, β coefficient (*P* value)	Self-rated health, β coefficient (*P* value)		ADL^b^, β coefficient (*P* value)	iADL^c^, β coefficient (*P* value)	Cognition, β coefficient (*P* value)
Internet use level	0.045 (.40)	0.048 (.45)	0.012 (.83)	–0.166 (.008)	–0.022 (.38)		0.004 (.86)	–0.018 (0.47)	–0.148 (<.001)	–0.079 (<.001)		–0.012 (=.59)	–0.010 (=.64)	–0.110 (<.001)
Control	Yes	Yes	Yes	Yes	Yes		Yes	Yes	Yes	Yes		Yes	Yes	Yes
*R* ^2^	0.264	0.156	0.181	0.081	0.176		0.093	0.138	0.052	0.149		0.090	0.127	0.036

^a^The numbers in the table represent the standardized regression coefficients for each submodel.

^b^ADL: activity of daily living.

^c^iADL: instrumental activity of daily living.

## Discussion

### Principal Findings

This study investigates the impact of internet access and use on health disparities among older adults in China, using multiple linear regression analysis and validating the findings with PSM methods. The results indicate that, in 2018, internet access and use tended to widen health inequalities among older adults, whereas in 2020, the effects were found to mitigate these inequalities. Factors such as urban or rural residence, sex, age, and education level also influenced the extent of these effects.

First, dual inequalities were observed in both the digital divide and health disparities among older adults. Significant improvements in internet access and use were noted between 2018 and 2020. In 2020, broadband coverage among older adults’ households reached 57.29% (6974/12,173), the proportion of older adults owning smart devices was 35.5% (4321/12,173), and 3.3% (402/12,173) owned multiple digital devices. In contrast, these figures were 39.22% (2998/7644), 10.5% (803/7644), and 1.98% (152/7644), respectively, in 2018. Regarding health indicators, the proportion of older adults rating their health as “very good,” along with ADL, iADL, and cognitive ability scores, all changed between 2018 and 2020, showing mixed trends. There was a slight improvement in self-rated health and ADL scores, whereas iADL and cognitive ability scores declined.

Second, results showed that internet access and use had differing impacts on health inequalities among older adults in 2018 and 2020. Our analysis of the 2018 data revealed that internet access and use generally exacerbated health inequalities for older adults. Specifically, internet access worsened disparities in subjective self-rated health, ADL, and cognition. However, it improved health inequalities in iADL. Through the internet, people could more conveniently handle daily affairs, reducing their reliance on physical services or facilities to a certain extent and contributing to the enhancement of iADL levels. Particularly during the COVID-19 pandemic, services such as online shopping and telemedicine enabled individuals to complete many daily activities at home, minimizing outdoor activities and the risk of infection. This trend may have, to some degree, ameliorated health inequalities related to iADLs. Furthermore, iADLs may be more sensitive to changes in older adults’ functional abilities, and even at lower levels of internet access, it could sensitively detect improvements in health equity. Furthermore, while the use of the internet appeared to have mitigated the disparity in iADL performance and cognitive abilities, it significantly intensified the imbalance between ADL performance and subjective health assessments. These findings remained robust after conducting a PSM test. This is consistent with previous research that used 2018 data from the Norwegian Diabetes Association to demonstrate that the digital divide can maintain and create health inequalities [61], which is basically consistent with our research results.

It was interesting to note that the results from 2020 showed that internet access and use actually contributed to improving health inequalities among older adults. Specifically, internet access had a mitigating effect on disparities in self-rated health, ADL, iADL, and cognitive inequality. In addition, internet use also narrowed inequalities in subjective self-rated health, ADL, iADL, and cognition. These findings remained robust following a PSM test. A 2020 study focusing on Chinese older adults found that digital participation played a mediating role in reducing depressive risks by indirectly improving mental health through the promotion of healthy lifestyles [62]. During the use of the internet, interaction and communication could stimulate thinking and broaden horizons, potentially mitigating differences in cognitive abilities to a certain extent. Meanwhile, given the severe aging issue in China, with many older individuals living alone, their children and friends could provide more timely care through mobile phones, which could reduce feelings of loneliness and benefit mental health among older adults.

During the COVID-19 pandemic in 2020, social distancing and travel restrictions prompted older adults to rely more heavily on the internet for socializing, shopping, and telemedical consultations, with the scale of China’s mobile health care users reaching 661 million. This trend led to an increase in internet use. Furthermore, the COVID-19 pandemic itself could impact health inequalities among older adults. During this period, older adults faced greater psychological pressure and loneliness, adverse to cognitive function, while psychological panic might also introduce bias in self-assessments of health status. The inability to undergo physical examinations in person due to lockdowns and other restrictions negatively impacted their physical health. Older adults with lower ADL scores might encounter greater challenges in maintaining their daily living activities. However, internet use also provided conveniences for older adults, offering easier access to services and resources necessary for daily life, such as online shopping and remote household services. Older adults could better manage their daily lives using online calendars, reminder functions, and similar tools. In addition, they could access health information and participate in online health courses to support their health management.

Third, the difference in results between 2018 and 2020 can be attributed to the different stages of internet access, which had different effects on health equity. During the initial stages, as observed in 2018, the rate of internet access was low, potentially leading to widened health disparities among older adults due to their internet access and use. This was primarily because there was a learning curve associated with adopting new technologies, which was particularly challenging for older adults to master.

As internet technology became more widespread and society began to pay more attention to older adults’ internet use, as seen in 2020, the gap in health inequalities started to diminish. The internet has been evolving with simpler and more intuitive designs, making it easier for older adults to navigate. Society has also been offering more internet education and training resources to help older adults improve their digital skills. For instance, in 2019, the Chinese government issued relevant policies to promote healthy aging. Furthermore, the range of health information and services available on the web has been expanding, including telemedicine and online health management. These services have become more accessible to older adults, regardless of their socioeconomic status [63]. These factors have all been working together to reduce the disparity in internet use among older adults, ultimately leading to a more equitable health landscape for this demographic.

Fourth, this study examines the heterogeneity of internet access and use on health inequalities in older adults concerning urban and rural areas, sex, age, and education level. In the older adult population, the influence of internet access on mitigating health inequalities is more pronounced among rural residents, although it also has a significant effect on urban dwellers. This could be explained by the fact that older adults in rural areas, through internet connectivity, gain access to health resources that were previously unavailable to them, whereas older adults in urban areas, who have had such access before, may experience less significant changes. However, older adults in urban areas tend to benefit more from acquiring and using the internet, as it facilitates improved health care information and services as well as living conditions. Conversely, older adults in rural areas may encounter greater life pressures and health challenges, coupled with a scarcity of resources and skills necessary for effective internet use. Consequently, these 2 groups exhibit varying degrees of impact on health outcomes. In terms of urban and rural areas, internet access has a more substantial impact on reducing health inequality among rural residents, who have limited access to health information. Accessing health information through the internet is crucial for improving the health of older adults in rural areas. Regarding sex, the effects of internet access on health inequalities are similar for men and women, whereas the effects of internet use are more prominent in men, possibly due to their greater aptitude for learning about electronics compared to women. In terms of age, the impact of internet access on reducing self-rated health inequalities and cognitive ability is more significant among younger adults aged <60 years, while the effect of internet use on both is essentially the same. Older adults approaching retirement tend to have stronger cognitive abilities and find it easier to learn how to use digital devices and online skills, resulting in a more significant impact. Regarding education level, internet access has a greater impact on self-rated health among the highly educated population. Subjective self-assessment of health is an individual's personal evaluation, which is more influenced by the health knowledge they acquire. The application of science and technology promotes the popularization of health knowledge [64]. Highly educated individuals generally pay more attention to their health conditions and possess better information literacy [65,66]. Consequently, their subjective self-evaluation of health becomes more distinct from that of the unconnected population.

Finally, although the initial impact of internet access and use on health inequalities in older adults tends to increase before decreasing, this observation does not imply that we can neglect inequalities during the early stages. Instead, we should focus more on addressing the challenges and barriers that older adults face when initially using the internet. Through policy guidance, social support, and ongoing technological innovation, we can promote more equitable access for older adults to the health and well-being benefits that the internet provides. On the basis of this understanding, the following policy recommendations are proposed:

- Expand internet coverage to enable older adults to benefit more from digital resources.
- Promote active aging by enhancing older adults’ information literacy, including training on digital skills through community programs and volunteerism.
- Strengthen internet coverage in rural areas to improve access to health information.
- Encourage collaboration between different sectors to address multiple social inequalities, such as interdepartmental cooperation to provide financial and technical support for the care of older adults.

There are several limitations to this paper. First, this study only used cross-sectional data from 2018 and 2020 to analyze the relationship between digital access and use and health outcomes among older adults, which limits our ability to establish causality and introduces the potential for reverse causality bias. Further longitudinal analysis is needed to assess the long-term effects and clarify the temporal sequence of these associations. Second, while this research attributes the difference in the impact of the internet on health inequality between 2018 and 2020 to varying levels of internet access, the specific impact mechanism and regulatory effects require additional verification in subsequent steps. Finally, although we conducted extensive stratified analyses across regions, education levels, urban and rural status, sex, and age, and included a wide range of control variables, we did not use survey weights. This choice was made to focus on associations rather than population-level inference, but it may affect the representativeness of our findings.

### Conclusions

This study examined the relationship between internet access and use on health inequalities among older adults, finding that their impact initially widened and then narrowed these inequalities. As internet technology became more widespread and advanced, increased social attention and support for older adults’ digital engagement contributed to this reduction. The internet is a powerful tool for improving health equity, but older adults often face barriers such as age and educational level, which make access to digital health benefits more challenging. Particularly, older adults in rural areas and with lower education levels have less access to information, making internet access even more crucial for improving health outcomes in these populations. Governments and society can promote equal, safe, and effective access to the health benefits of the internet for older adults through policy, social support, and technological innovation.

## Appendix

Multimedia Appendix 1 Common support and balance test.

## Abbreviations

ADL: activity of daily living
CHARLS: China Health and Retirement Longitudinal Study
iADL: instrumental activity of daily living
MMSE: Mini-Mental State Examination
PSM: propensity score matching

## Data Availability

The datasets generated and analyzed during this study are available in the China Health and Retirement Longitudinal Study data repository [67].
