# Supplementary material for: The Association Between the Digital Divide and Health Inequalities Among Older Adults in China: Nationally Representative Cross-Sectional Survey
Source: J Med Internet Res. 2025 Jan 15;27:e62645. doi: 10.2196/62645 (PMC11780301; doi:10.2196/62645)
Supplement: Multimedia Appendix 1 [file jmir_v27i1e62645_app1.docx]

## Appendix 1. Common support and balance test

The trend of the experimental group and the control group samples before and after matching is verified by the kernel density function, and the results of the kernel density function before (left panel) and after (right panel) matching are shown in the attached Figures 1-4. The Figures show that the kernel density function trend graphs of the two subsamples after matching are more convergent, i.e., this paper uses the PSM model to reduce the distributional difference of the variables between the two subsamples, and eliminates the self-selection of the sample to some extent.


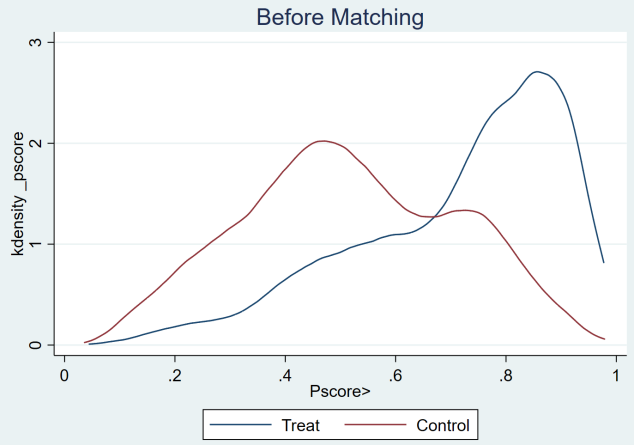

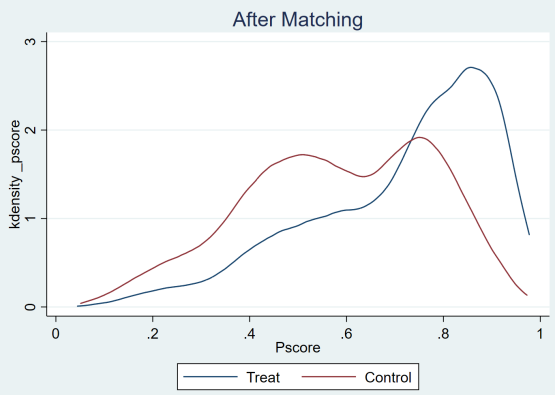


Attached Figure 1. Pre-match kernel density map (left) vs. post-match kernel density map (right) for Internet access in 2020.


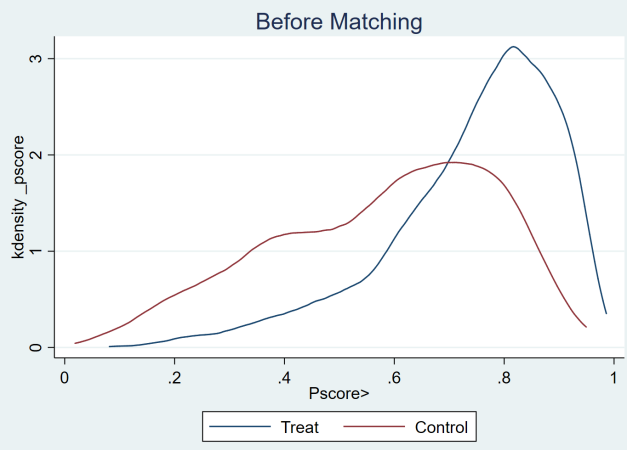

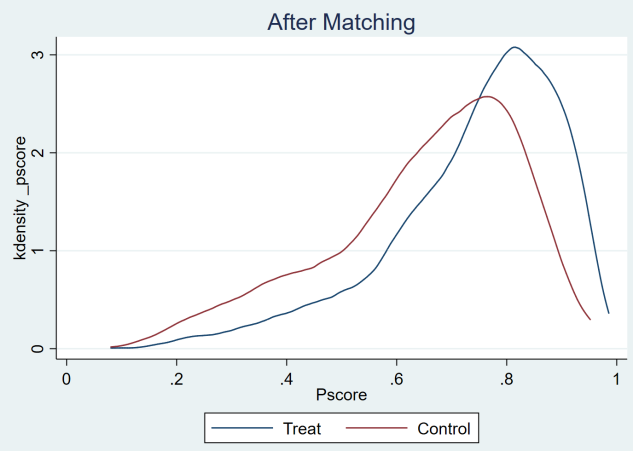


Attached Figure 2. Pre-match kernel density map (left) vs. post-match kernel density map (right) for Internet use in 2020.


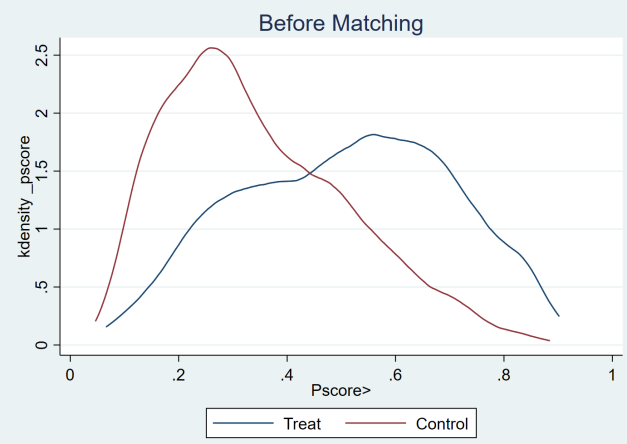

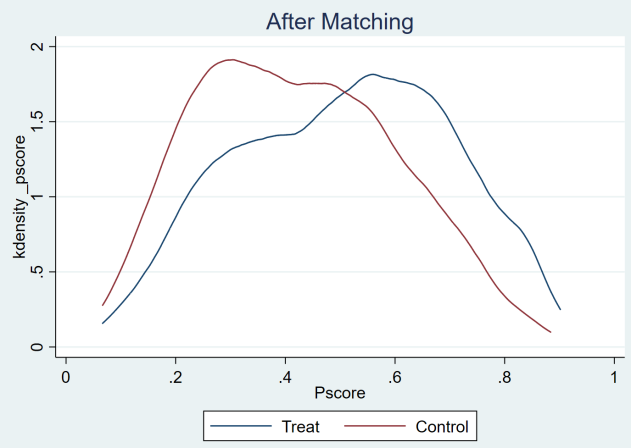


Attached Figure 3. Pre-match kernel density map (left) vs. post-match kernel density map (right) for Internet access in 2018.


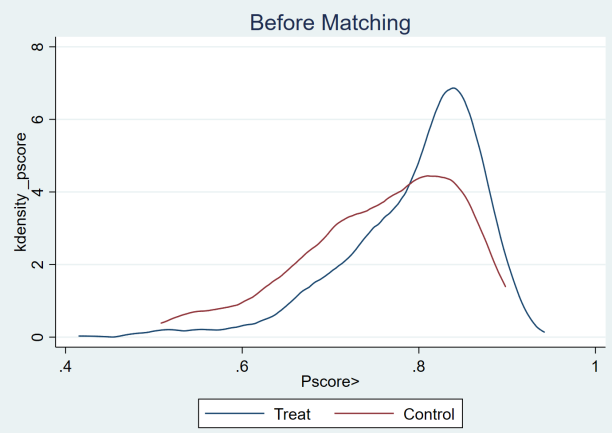

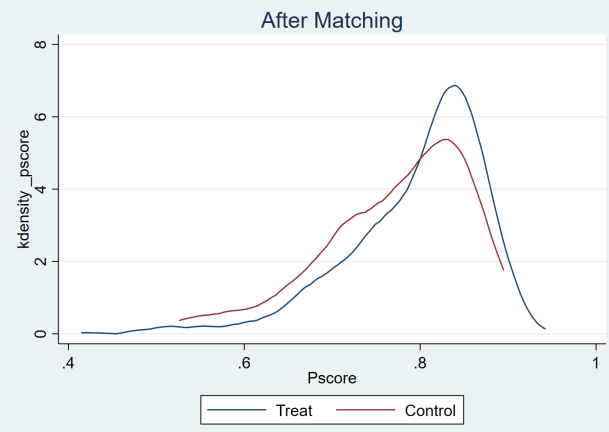


Attached Figure 4. Pre-match kernel density map (left) vs. post-match kernel density map (right) for Internet use in 2018

Attached Table 1. Balance test of all samples.

| variables | 2018 | | | | |  | 2020 | | | | |
| --- | --- | --- | --- | --- | --- | --- | --- | --- | --- | --- | --- |
|  | Match type | Bias | Reduced bias (%) | t Value | *P* |  | Match type | Bias | Reduced bias (%) | t Value | *P* |
| Male | Unmatched | -0.4 |  | -0.15 | 0.88 |  | Unmatched | 7.2 |  | 3.63 | <0.001 |
|  | Match | 2.2 | -539.4 | 0.89 | 0.376 |  | Match | 1.0 | 85.6 | 0.62 | 0.536 |
| Age | Unmatched | -55.4 |  | -23.78 | <0.001 |  | Unmatched | -74.5 |  | -37.84 | <0.001 |
|  | Match | -2.5 | 95.4 | -1.04 | 0.299 |  | Match | -6.2 | 91.7 | -3.77 | <0.001 |
| Rural | Unmatched | -40.2 |  | -17.85 | <0.001 |  | Unmatched | -41.5 |  | -20.08 | <0.001 |
|  | Match | 5.5 | 86.4 | 1.83 | 0.067 |  | Match | 4.8 | 88.5 | 2.45 | 0.014 |
| Secondary schools | Unmatched | -18.8 |  | -8.09 | <0.001 |  | Unmatched | -17.1 |  | -8.68 | <0.001 |
|  | Match | 3.3 | 82.5 | 1.33 | 0.184 |  | Match | 6.6 | 61.6 | 4.00 | <0.001 |
| Junior high school and above | Unmatched | 54.1 |  | 23.71 | <0.001 |  | Unmatched | 62.8 |  | 30.57 | <0.001 |
|  | Match | -0.5 | 99.1 | -0.18 | 0.859 |  | Match | -7.1 | 88.7 | -3.75 | <0.001 |
| Married | Unmatched | 22 |  | 9.36 | <0.001 |  | Unmatched | 23.1 |  | 12.01 | <0.001 |
|  | Match | 7.9 | 64.3 | 3.23 | 0.001 |  | Match | 9.3 | 59.6 | 5.90 | <0.001 |
| Urban and rural resident medical insurance | Unmatched | -1.4 |  | -0.59 | 0.555 |  | Unmatched | -32.8 |  | -15.87 | <0.001 |
|  | Match | 1.2 | 11.1 | 0.49 | 0.623 |  | Match | 6.0 | 81.8 | 3.04 | 0.002 |
| Chronic | Unmatched | -2.5 |  | -1.05 | 0.292 |  | Unmatched | -1.2 |  | -0.62 | 0.533 |
|  | Match | 5.3 | -114.3 | 2.09 | 0.037 |  | Match | 1.1 | 9.4 | 0.67 | 0.503 |
| Disability | Unmatched | -12.3 |  | -5.21 | <0.001 |  | Unmatched | -26.0 |  | -13.74 | <0.001 |
|  | Match | -1.4 | 88.7 | -0.58 | 0.559 |  | Match | -4.1 | 84.3 | -2.79 | 0.005 |
| Outpatient visit | Unmatched | -1.3 |  | -0.57 | 0.570 |  | Unmatched | -0.7 |  | -0.37 | 0.713 |
|  | Match | 0.4 | 71.7 | 0.15 | 0.883 |  | Match | 2.7 | -279.5 | 1.65 | 0.098 |
| Intergenerational support | Unmatched | 34.1 |  | 14.55 | <0.001 |  | Unmatched | -11.7 |  | -5.94 | <0.001 |
|  | Match | 4.6 | 86.6 | 1.89 | 0.059 |  | Match | 1.5 | 87.2 | 0.90 | 0.367 |
| Log(income) | Unmatched | 15.5 |  | 6.61 | <0.001 |  | Unmatched | 15.1 |  | 7.72 | <0.001 |
|  | Match | 0.5 | 96.7 | 0.21 | 0.830 |  | Match | 5.0 | 67.0 | 3.11 | 0.002 |
| Central Region | Unmatched | 3.5 |  | 1.52 | 0.130 |  | Unmatched | 5.9 |  | 2.97 | 0.003 |
|  | Match | 0.3 | 92.2 | 0.11 | 0.914 |  | Match | 6.1 | -3.0 | 3.63 | <0.001 |
| Western Region | Unmatched | -7.6 |  | -3.27 | 0.001 |  | Unmatched | -10.0 |  | -5.09 | <0.001 |
|  | Match | -1.6 | 79.7 | -0.62 | 0.536 |  | Match | -1.4 | 86.4 | -0.82 | 0.410 |
| Northeast Region | Unmatched | 2.8 |  | 1.21 | 0.226 |  | Unmatched | 8.4 |  | 4.15 | <0.001 |
|  | Match | -1.4 | 49.0 | -0.54 | 0.586 |  | Match | -9.4 | -12.6 | -4.89 | <0.001 |

## Appendix 2. The sort of the region

Attached Table 1. The sort of the region.

| **Region** | **Province (autonomous region/municipality）** |
| --- | --- |
| Eastern Region | Beijing, Tianjin, Hebei, Shanghai, Jiangsu, Zhejiang, Fujian, Shandong, Guangdong, Hainan |
| Central Region | Shanxi, Anhui, Jiangxi, Henan, Hubei, Hunan |
| Western Region | lnner Mongolia, Guangxi, Chongqing, Sichuan, Guizhou, Yunnan, Tibet, Shaanxi, Gansu, Xinjiang, Qinghai |
| Northeast Region | Liaoning, Jilin, Heilongjiang |
